# Recent Advances in miRNA-Based Therapy for MASLD/MASH and MASH-Associated HCC

**DOI:** 10.3390/ijms252212229

**Published:** 2024-11-14

**Authors:** Sara Carpi, Simona Daniele, Jacqueline Fátima Martins de Almeida, Daniela Gabbia

**Affiliations:** 1 Department of Health Sciences, University ‘Magna Græcia’ of Catanzaro, 88100 Catanzaro, Italy; 2 NEST (National Enterprise for nanoScience and nanoTechnology), Istituto Nanoscienze-CNR and Scuola Normale Superiore, 41125 Modena, Italy; 3 Department of Pharmacy, University of Pisa, 56126 Pisa, Italy; simona.daniele@unipi.it (S.D.); jacqueline.moraes@farm.unipi.it (J.F.M.d.A.); 4 Department of Pharmaceutical and Pharmacological Sciences, University of Padova, 35131 Padova, Italy

**Keywords:** metabolic dysfunction-associated steatotic liver disease, metabolic dysfunction-associated steatohepatitis, hepatocellular carcinoma, microRNA, tumorigenesis, miRNA-based therapy

## Abstract

Metabolic dysfunction-associated steatotic liver disease (MASLD), formerly known as non-alcoholic fatty liver disease (NAFLD), is a growing health concern worldwide, affecting more than 1 billion adults. It may progress to metabolic dysfunction-associated steatohepatitis (MASH), cirrhosis, and ultimately hepatocellular carcinoma (HCC). Emerging evidence has demonstrated the role in this transition of microRNAs (miRNAs), which regulate the expression of genes associated with lipid metabolism, inflammation, fibrosis, and cell proliferation. Specific miRNAs have been identified to exacerbate or mitigate fibrotic and carcinogenic processes in hepatic cells. The modulation of these miRNAs through synthetic mimics or inhibitors represents a promising therapeutic strategy. Preclinical models have demonstrated that miRNA-based therapies can attenuate liver inflammation, reduce fibrosis, and inhibit tumorigenesis, thus delaying or preventing the onset of HCC. However, challenges such as delivery mechanisms, off-target effects, and long-term safety remain to be addressed. This review, focusing on recently published preclinical and clinical studies, explores the pharmacological potential of miRNA-based interventions to prevent MASLD/MASH and progression toward HCC.

## Simple Summary

Metabolic dysfunction-associated steatotic liver disease (MASLD), formerly known as non-alcoholic fatty liver disease, affects over one billion adults worldwide. It can progress to more severe conditions, like metabolic dysfunction-associated steatohepatitis (MASH), cirrhosis, and hepatocellular carcinoma (HCC). Recent studies have highlighted the role of microRNAs (miRNAs) in this progression due to their ability to regulate genes involved in lipid metabolism, inflammation, fibrosis, and cell proliferation. Modulating miRNAs through synthetic molecules represents a promising therapeutic strategy. Preclinical models demonstrate that miRNA-based therapies can reduce liver inflammation and fibrosis and inhibit tumorigenesis, potentially delaying or preventing HCC onset. However, challenges such as delivery mechanisms, side effects, and long-term safety remain to be addressed. This review, focusing on recent preclinical and clinical studies, explores the pharmacological potential of miRNA-based interventions to counteract MASLD/MASH and their progression to HCC. It provides insights into an emerging field with significant implications for liver disease treatment.

## 1. Introduction

Since its first characterization in 1980 by Ludwig and colleagues, non-alcoholic fatty liver disease (NAFLD) has been defined as an accumulation of more than 5–10% of hepatic fat without other co-existing secondary causes, like significant alcohol consumption (more than 21 drinks/week in men and more than 14 drinks/week in women), steatogenic drugs, or other inherited or acquired hepatic metabolic states [1]. In 2023, after the multi-society Delphi statement, NAFLD was renamed as metabolic dysfunction-associated steatotic liver disease (MASLD), and the diagnostic criteria were strictly redefined in order to avoid patients’ stigmatization and improve diagnosis and therapy [2]. MASLD is diagnosed by hepatic steatosis, as with NAFLD, in addition to one or more of five cardiometabolic risk factors (CMRFs). Additionally, a new category was termed metabolic and alcohol related/associated liver disease (MetALD), including those patients with MASLD who consume alcohol more than 140–350 g/week and 210–420 g/week for women and men, respectively. Metabolic dysfunction-associated steatohepatitis (MASH), the advanced stage of MASLD, has become a major cause of advanced fibrosis, cirrhosis, and hepatocellular carcinoma (HCC), the third cause of cancer-related death [3]. As a consequence of the obesity pandemic and the widespread prevalence of type 2 diabetes, an increase in the number of MASH patients worldwide is expected in the future. In March 2024, the first drug for NASH patients was approved by the Food and Drug Administration (FDA), marking a significant milestone in the treatment of this pathology [4], but the global disease burden and the great impact on public health costs are fueling the development of well-tolerated, more effective, and safe pharmacological treatments [3]. MASH could lead to advanced fibrosis and liver cirrhosis and is becoming one of the two main primary causes of HCC globally with hepatitis [5,6,7]. Traditionally, HCC formation has been considered to evolve from MASH through a multi-stage process of inflammation, fibrosis, and cirrhosis; however, recent research has found that steatohepatitis may progress directly to HCC without fibrosis and cirrhosis [8,9,10,11]. Thus, there is a huge need for effective treatment that can prevent its cancerous progression.

In the dysregulated pathways that promote the progression of MASLD to HCC, microRNAs (miRNAs) play an important role due to their ability to regulate lipid metabolism, glucose homeostasis, cell proliferation, apoptosis, migration, and differentiation [12]. In recent decades, many miRNAs have been investigated as markers for the diagnosis of chronic liver diseases, in particular HCC, in order to set up a strategy for early diagnosis and therapy outcome prediction [13,14,15]. In fact, MASH is the strongest predictor of fibrotic progression in MASLD and has become one of the two main causes of tumorigenic evolution [9].

miRNAs, non-coding single-stranded RNA strands of 20–25 nucleotides, play a role in a variety of physiological and pathological pathways by regulating the expression of downstream genes. In recent years, many studies have suggested that they play a role in the pathogenesis of NAFLD/NASH by controlling disease development and progression at various levels [16]. They are likely to represent the epigenetic modifications most extensively studied in NAFLD, and their role in hepatocarcinogenesis has also been reviewed [17]. The modulation of these miRNAs through synthetic mimics or inhibitors represents a promising therapeutic strategy that is gaining increasing attention in basic and clinical research.

This review provides an overview of the most promising miRNAs that are able to exacerbate or mitigate liver fibrotic and carcinogenic processes. This review also summarizes preclinical and clinical studies investigating the pharmacological potential of miRNA-based interventions to counteract MASLD/MASH and prevent progression to HCC.

## 2. Role of miRNAs in the Pathophysiology of MASLD/MASH and HCC Progression

Steatotic liver disease (SLD) comprises a spectrum of subcategories, including, among others, MASLD, MetALD (MASLD with increased alcohol intake), and MASH [2,18]. The recently approved change in NAFLD nomenclature into MASLD and the better subcategorization of SLD have garnered much attention in the field of hepatology and metabolic research. The new diagnostic criteria can more accurately identify people at higher risk of diabetes [18,19]. Previously, evidence showed that NAFLD has a 25% prevalence worldwide, reaching 30% in the USA and up to 32.9% in China. These patients are at risk of developing NASH, and 20% of NASH patients can progress to cirrhosis, with a higher risk of HCC [19]. These prevalence data overlap substantially with the most recent data on the global burden of MASLD/MASH [5]. Metabolic syndrome, along with associated factors, such as insulin resistance, oxidative stress, lipid peroxidation, proinflammatory cytokines, lipotoxicity, endoplasmic reticulum stress, adipose tissue, gut microbiota, and genetics, significantly contribute to the pathogenesis and progression of MASLD and MASH, although our understanding of this disease remains limited due to its broad range and complexity [20,21,22,23]. To better clarify the pathophysiology of MASLD/MASH and its relation to HCC progression, understanding the role of miRNAs in this process offers a promising avenue.

In the human genome, only approximately 1–2% of total DNA is associated with known protein-coding transcripts; these data have been confirmed through comparative genomics, transcriptional studies, and the complete sequencing of the human genome [17]. Moreover, these studies have revealed that only a small fraction of the genome encodes functional proteins, while the majority of the DNA serves other functions, including gene expression regulation and the production of non-coding RNA molecules. Thirty years ago, researchers discovered that genomic components traditionally regarded as nonfunctional could regulate gene expression [24]. Indeed, a variety of non-coding RNAs, both short and long, have evolved in eukaryotes to exert control over genetic materials and transcripts [25,26].

miRNAs function as negative regulators of gene expression and are one of the most conserved classes of small non-coding RNAs [27]. Human precursors miRNAs, which have the primary function of inhibiting the expression of genes, are estimated to number over 2000 in the human genome [28]. However, it is crucial to recognize that research in this area is continuously evolving, and new miRNAs are being identified regularly. Scientists are constantly discovering novel miRNAs with diverse functions and roles in various biological processes.

miRNAs are formed from miRNA precursors (pri-miRNAs), which are several hundred nucleotides long and are synthesized by the RNA polymerase II enzyme within the cell nucleus [29]. Pri-miRNAs, characterized by stem–loop structures, are identified by the RNase III family enzyme Drosha and its double-stranded RNA binding protein, DiGeorge Syndrome Critical Region 8 (DGCR8). In the next step, Drosha cleaves the pri-miRNA to produce pre-miRNA, which has a stem–loop structure and is 70 nucleotides in length. This pre-miRNA is then transported to the cytoplasm by exportin-5. In the cytoplasm, pre-miRNAs are processed by the ribonuclease Dicer into a double-stranded miRNA molecule (miRNA-miRNA* duplex) consisting of driver and passenger strands; each strand is 20–21 nucleotides long. As mature miRNAs, either strand can potentially act as an active miRNA, but typically only one, known as the guide strand, is incorporated into miRNA-Induced Silencing Complexes (miRISCs). The passenger strand (miRNA*) is usually rapidly degraded [27].

**Figure 1:**
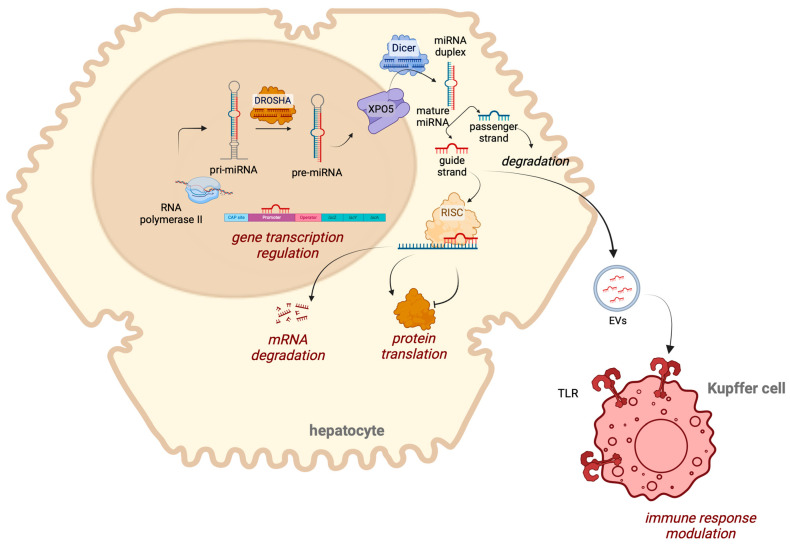
miRNA biogenesis and functions. RNA polymerase II transcribes pri-miRNA in the nucleus, which is processed by DROSHA into pre-miRNA. The pre-miRNA is exported to the cytoplasm via XPO5, where it is further processed into a mature miRNA duplex. The guide strand of the miRNA associates with the RISC complex to regulate mRNA degradation or inhibit protein translation. miRNAs can directly interact with a DNA promoter or regulatory sequence to modulate gene transcription. Additionally, miRNAs can be released in extracellular vesicles and interact with TLR receptors to modulate the immune response. Abbreviations: EVs, extracellular vesicles; pre-miRNA, precursor miRNA; pri-miRNA, primary miRNA; RISC, RNA-induced silencing complex; TLR, Toll-like receptor; XPO5, exportin 5. Created in BioRender.

It has been noted that miRNAs play several roles in regulating gene expression, including repressing mRNA at the post-translational level [30]. The miRISC complex generally binds to the 3ʹ untranslated regions (UTRs) of specific target mRNAs (Figure 1), leading to reduced protein expression and enhanced degradation in the target mRNA. In particular, one miRNA has the capacity to bind hundreds of 3′-UTRs of different mRNAs, and conversely, one mRNA can be targeted by multiple miRNAs. Recent research has shown that miRNAs can also interact with ribonucleoproteins independently of RISC, disrupting their RNA binding capabilities, a process known as decoy activity [31]. Furthermore, some studies have suggested that miRNAs can directly affect gene transcription by binding to promoter or regulatory regions of DNA [32]. Finally, other mechanisms have been described for miRNAs, including direct binding to Toll-like receptors, or mitochondrial transcripts, which further outline our currently limited understanding of miRNA biology [16].

**Figure 2:**
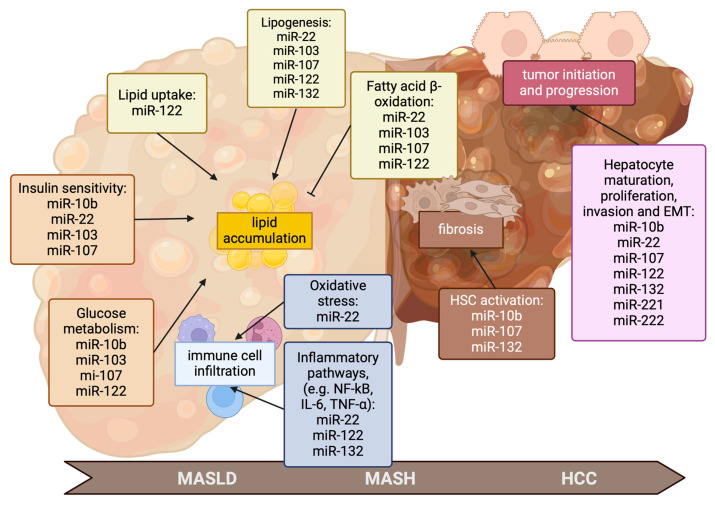
Role of miRNAs in the pathophysiology of MASLD/MASH and HCC progression. Different cellular and metabolic processes affected by miRNAs involved in the progression of MASLD/MASH and HCC, including insulin resistance, oxidative stress, lipid peroxidation, proinflammatory cytokines, lipotoxicity, endoplasmic reticulum stress, adipose tissue, gut microbiota, and genetics, significantly contribute to the pathogenesis and progression of MASLD and MASH. Abbreviations: EMT, epithelial–mesenchymal transition; IL-6, interleukin 6; miR, microRNA; NF-kB, nuclear factor kappa B; TNF-α, Tumor necrosis factor-alpha. Blunt arrows (┴) indicate inhibition, while sharp arrows (→) indicate stimulation. Created in BioRender.

miRNAs regulate essential biological processes that include the trafficking, anabolism, and catabolism of lipids in hepatic cells. Furthermore, miRNAs control other pathways that contribute to the development of steatosis, such as carbohydrate metabolism and stress-activated pathways. All these metabolic processes are closely regulated by specific miRNAs that have been exploited as targets in the development of new therapeutic approaches, as summarized in Figure 2 [16]. Despite the high complexity of miRNA biology, several of them have already been identified as important actors in liver diseases, and miRNA-based therapies are in development.

Recent research has identified abnormal levels of miRNAs in the liver and bloodstream of patients with MASH and HCC. Thus, a simple method of measuring circulating miRNAs could serve as diagnostic and prognostic marker, and possibly as a therapeutic target for MASH/HCC in a large population with MASLD. Although miRNA dysregulation may contribute to disease pathogenesis, the exact role and mechanisms remain unclear, particularly in MASH-related hepatocarcinogenesis. In tumor tissues, microRNAs that promote cancer development and progression (oncomiRs) and those involved in the promotion of metastasis (metastamiRs) are overexpressed, while microRNAs that induce apoptosis (apoptomiRs) and tumor-suppressive-miRNAs are typically downregulated, and other miRNAs may contribute to chemoresistance and immune evasion [33]. Detecting abnormal miRNA expression in MASH or HCC alone does not confirm its role in disease development, as it could be a secondary effect. To establish a link between miRNA alterations and MASH-related HCC, it is necessary to demonstrate that there is a mechanistic connection between miRNA alterations and HCC development, and miRNA changes occur in the preneoplastic stages, persist, and accumulate as HCC progresses [34]. An analysis in steatotic, NASH, and HCC mice revealed 10 differentially expressed miRNAs in NASH and HCC with respect to the controls, including miR-107-3p, miR-221-3p, miR-222-3p, and miR-223-3p, that are involved in major liver carcinogenesis-related pathways, e.g., cell cycle regulation, stem cell regulation, epithelial–mesenchymal transition (EMT), TGF-β, STAT3, Wnt/β-catenin, ERK/MAPK, PPARα/RXRα, PTEN, mTOR, and NF-κB signaling [34].

miR-122, the most abundant microRNA in the human liver, comprises more than 70% of the total liver miRNA pool [35]. It plays a crucial role in hepatocyte maturation by stimulating the expression of 24 hepatocyte-specific genes, including hepatocyte nuclear factor 6 (HNF6). miR-122 is closely associated with both glucose and lipid metabolism, and its inhibition has been shown to reduce plasma cholesterol levels and improve liver steatosis, with no adverse effects observed from downregulating miR-122 in the liver [36]. Moreover, it can reduce lipogenesis, downregulating the lipogenic enzymes fatty acid synthase (FAS) and acetyl-CoA carboxylase (ACC), reduce lipid uptake from circulation and intracellular triglyceride accumulation, and increase fatty acid β-oxidation, all mechanisms involved in the onset of MASLD [37]. Interestingly, circulating miR-122 results increased in MASLD/MASH after secretions from the liver into the circulation due to a compensative mechanism involving hepatic free fatty acid sensing [38,39]. miR-122 is also involved in the regulation of inflammatory pathways, e.g., NF-kB, and the transfection of miR-122 inhibitor into an NAFLD cell model counteracted the secretion of proinflammatory cytokines due to the activation of the TLR4/MyD88/NF-κBp65 signaling pathway [40]. The overexpression of miR-122 can suppress cell proliferation and increase the chemosensitivity of cancer cells to antitumor drugs [41,42]. Therefore, the restoration of miR-122 levels can lower the incidence of HCC, suggesting a potential therapeutic approach to treating HCC by increasing miR-122 expression, although no clinical trials have been undertaken so far [43].

Another miRNA that has been investigated for obesity and liver steatosis is miR-22. In a mouse model of diet-induced obesity (DIO), the administration of antago-miR-22-3p significantly reduced fat mass, improving metabolic parameters by acting on adipocytes, increasing mitochondrial mass and activity and UCP1 expression [44]. The subcutaneous administration of the antago-miR-22-3p APT-110 effectively reduced insulin sensitivity and circulating levels of glucose and cholesterol, thus suggesting that the form 3p also plays a role in MASLD/MASH development as well as in cancer [45,46]. This effect is associated with a reduction in plasma leptin, an adipokine involved in long-term energy balance, and an increase in energy expenditure that is likely to be correlated to an increased amount of thermogenic adipose tissue [45]. Similar results have been obtained in genetic murine models, in which miR-22 knockout protected against obesity and hepatic steatosis, while its overexpression promoted fat accumulation and steatosis even with a standard diet [47]. One of the critical mechanisms in MASLD/MASH progression is increased oxidative stress, which leads to hepatocyte apoptosis that releases apoptotic bodies able to activate inflammatory response in Kupffer cells (KCs), the resident hepatic macrophages, and to induce hepatic stellate cell (HSC) activation, thus promoting fibrogenesis [48]. Treatment with a miR-22 inhibitor was shown to improve inflammation in steatotic L02 cells, by reducing the expression of proinflammatory factors, e.g., TNF-α and IL-6. In addition, it was able to reduce sterol regulatory element-binding protein 1 (SREBP-1), a transcription factor involved in lipid and cholesterol production, and concurrently upregulate Forkhead box protein O1 (Foxo1), involved in the regulation of gluconeogenesis and glycogenolysis [49]. Moreover, miR-22 overexpression was observed in male HCC-adjacent tissue and was associated with the downregulation of estrogen receptor α, which is likely to reduce the protective effect of estrogen, thus explaining the higher incidence of HBV-associated HCC in males compared to females [50]. In this context, antago-miR-22 may also exert a beneficial effect on tumorigenic progression in patients with MASH.

Many studies have observed that miR-132 increases in liver tissue from both animal NAFLD/NASH models and human patients. This miRNA finely tunes multiple targets involved in metabolic pathways, as well as in cell proliferation, epigenetic regulation, and intestinal inflammation [51]. It is able to modulate multiple pathways that control key enzymes involved in TG synthesis, e.g., pyruvate kinase isozyme L (L-PK), SREBP1, and 2 and FAS.

Among the emerging pharmacological targets for MASH, peroxisome proliferator-activated receptors (PPARs) have gained attention due to their ability to regulate insulin sensitivity, TG metabolism, fatty acid oxidation, fibrogenesis, and inflammation in the liver [52]. The pan-PPAR agonist lanifibranor has been shown to effectively reduce the SAF-A score (part of the Steatosis, Activity, Fibrosis scoring system) in a phase 2b trial, encouraging studies on PPAR agonists [3,53]. The regulatory networks of these nuclear transcription factors are complex and far from fully understood. In addition to ligands, co-regulators (coactivators/corepressors), and protein post-translational modifications (PTMs), miRNAs have also been shown to be able to regulate PPAR activation [54]. For example, PPAR-α could be directly regulated at the post-transcriptional level by miR-10b, and the transfection of anti-miRNA-10b into steatotic L02 cells significantly improved steatosis by acting on this pathway [55]. Furthermore, the administration of synthetic miR-10b-5p mimic to diabetic mice improved glucose homeostasis and gastrointestinal motility [56]. Interestingly, miR-10b expression increased in fibrotic and HCC livers of diethylnitrosamine (DEN)-treated rats accordingly to the worsening of fibrosis. These data suggest the role of this miRNA in the early phase of hepatic carcinogenesis [57]. Furthermore, the overexpression of miR-10b in HCC tissue has been demonstrated to sustain the migration and invasion of cancer cells by modulating RhoC, uPAR, MMP-2, and MMP-9 through the HOXD10 pathway [12]. The administration of miR-10b mimics was able to promote tumorigenesis in xenografted HCC mice by regulating the expression of CUB and Sushi multiple domains 1 (CSMD1), a protein involved in EMT [58].

Another miRNA exploited as a potential target for the MASLD/MASH/HCC transition is miR-103/miR-107. The suppression of miR-103/107 transcription resulted in an improvement in glucose homeostasis and insulin sensitivity due to the upregulation of caveolin-1 in adipocytes [59]. Furthermore, the administration of anti-miR-103 to *ob/ob* mice induced an increase in adiponectin levels, which could reduce TG in the muscle and liver due to the promotion of fatty acid combustion and energy dissipation [60,61]. A study conducted in 50 NAFLD patients demonstrated that a high expression of miR-103 increases adipogenesis in hepatocytes and leads to hypertriglyceridemia due to insulin resistance, thus promoting steatosis [62]. This evidence supports the role of miR103 in the pathogenesis of MASLD and insulin resistance. HSC activation plays a key role during fibrogenesis in the development of MASLD/MASH. Recently, it has been demonstrated that steatotic hepatocytes secrete miR-107-containing exosomes that could be shuttled to HSC, activating Wnt signaling by targeting Dickkopf-1 (DKK-1), and to CD4^+^ T lymphocytes, increasing interleukin 9 (IL-9) by targeting Forkhead box protein P1 (Foxp1). IL-9 further promotes HSC activation through the activation of the Raf/MEK/ERK pathway [63].

## 3. miRNA-Based Therapy in Preclinical and Clinical Development for MASLD/MASH

In the last decade, the field of miRNA-based therapy has seen significant advancements due to the ability of miRNAs to regulate mRNA expression. Dysfunctional miRNAs, observed in chronic liver diseases like MASLD/MASH and HCC, lead to the altered expression of inflammatory mediators, fibrotic factors, oncogenes, and tumor suppressors [64]. This has led to the development of synthetic miRNA mimics and inhibitors as innovative miRNA-based approaches. miRNA mimics, synthetic RNA duplexes that contain strands identical to the corresponding miRNAs, may restore or enhance miRNA functions, potentially correcting disrupted gene expression profiles in disease states. By replenishing specific miRNAs, these mimics can restore normal cell function and alleviate disease symptoms.

Conversely, the inhibition of miRNA can be achieved through the use of anti-miRNA oligonucleotides (anti-miRs) [65]. These single-stranded oligonucleotides, structurally similar to antisense oligonucleotides (ASOs), directly bind to target miRNAs, preventing their interaction with mRNA targets. This inhibition can suppress the pathological effects of overactive miRNAs, offering a promising utilization in miRNA-based therapy.

Currently, several miRNA-based therapies are in various stages of preclinical and clinical development (Table 1) [66]. The miR-122 antisense LNA (Miravirsen) was the first anti-miRNA oligonucleotide in clinical trials [67]. miR-122 inhibition has shown therapeutic relevance, with successful viral load reduction in HCV-infected primates and humans, leading to ongoing trials for miravirsen and RG-101 inhibitors. miR-122 mimics can also offer therapeutic benefits for NAFLD and HCC by preventing disease progression and reducing tumor aggressiveness, although this approach has not yet been clinically tested for MASH or HCC [16].

One clinical trial involving antago-miR-103a-3p/107 (AZD4076) has been completed, and another is active but not recruiting. A clinical trial for anti-miR-22-5p (RES-010, developed by Resalis Therapeutics) is expected to begin in 2024. Additionally, other miRNA-based therapies, such as anti-miR-132-3p (Regulus Therapeutics Inc., San Diego, CA, USA) and the miR-10b-5p mimic (RosVivo Therapeutics Inc., Reno, NV, USA), are currently in development [66].

These advances underscore the potential of miRNA-based therapies to offer novel and effective treatments for a wide range of diseases by specifically targeting and modulating gene expression pathways. As research progresses, the therapeutic applications of miRNA mimics and inhibitors are likely to expand, paving the way for new and innovative approaches to disease management.

### 3.1. microRNA-10b-5p

Recently, the microRNA miR-10b-5p has emerged as an interesting candidate in liver disease (Table 1). miR-10b-5p expression is reduced in diabetic patients and mice with insulin resistance [70,71]. Furthermore, serum levels of miR-10b-5p miR-10b were inversely correlated with the degree of inflammation in NASH patients [72].

miR-10b-5p targets the transcription factor Krüppel-like factor 11 (KLF11), which negatively regulates the expression of receptor tyrosine kinase (KIT). In 2021, Singh and colleagues showed that miR-10b-5p is a key regulator in diabetes and gastrointestinal dysmotility via the KLF11-KIT pathway. Researchers have demonstrated that injections of miR-10b-5p mimics improve glucose homeostasis and GI motility in mice in preclinical studies [56].

RosVivo Therapeutics Inc. has developed a miR-10b-5p mimic named RSVI-301. In some media communications, RosVivo Therapeutics Inc. has reported collecting strong and compelling data on the efficacy of RSVI-301 compared to the GLP-1 receptor agonist in animal models. As a result, Eli Lilly and Company is now involved with the miRNA mimic RSVI-301, and a phase 1 clinical trial for NAFLD is expected to launch in the next few years [73].

### 3.2. microRNA-132-3p

One potential therapeutic target identified for NAFLD/NASH is miR-132-3p (Table 1). Hepatic miR-132 levels are elevated in mouse models of alcoholic liver disease and in the hepatocytes of NASH patients [69,74]. Furthermore, conditional miR-132-overexpressing transgenic mice exhibit increased hepatic miR-132 and reduced metabolism-related miR-132 targets [51]. In particular, miR132 targets such as phosphatase and tensin homolog (Pten), Forkhead box O3 (FOXO3), and sirtuin 1 (Sirt1) are associated with hepatic steatosis, hyperlipidemia, and glucose regulation. The inhibition of miR-132 in diet-induced obese mice decreased hyperlipidemia and hepatic steatosis, showing a more significant effect compared to the partial impact of targeting individual miR-132 targets [51].

In April 2019, Regulus Therapeutics Inc. presented a late-breaking poster at the International Liver Congress of the European Association for the Study of the Liver (EASL) on the development of its lead anti-miR-132 treatment for NASH [69]. The lead candidate showed improvements in key endpoints in multiple animal models of NASH, including hyperglycemia, disease-related gene expression, liver transaminases, and NAFLD Activity Score (NAS). In the Amylin mouse model (a diet-induced NASH model), two to four weekly doses resulted in an early improvement in multiple disease parameters, such as liver triglycerides and blood transaminase levels. Eight weeks of treatment with anti-miR-132 induced significant improvements in liver fibrosis and hyperglycemia compared to the control group [69]. Unfortunately, after this point, no further official information was available on the development of the anti-miR-132 treatment by Regulus Therapeutics Inc.

### 3.3. microRNA-22

In the last decade, another miRNA, miR-22-5p, has emerged as a target in hepatic liver diseases. In humans, serum levels of miR-22-5p are reported to increase significantly in NAFLD patients and also in NAFLD patients undergoing fibrate therapy compared to controls. In an in vitro model of human HepG2 cells, miR-22-5p was induced and secreted into the culture medium after incubation with model steatotic drugs (valproate, doxycycline, cyclosporin A, and tamoxifen) [75].

Mechanistically, miR-22 directly targets crucial genes involved in regulating metabolism and obesity, such as the peroxisome proliferative-activated receptor (Pgc-1α), the peroxisome proliferator-activated receptor α (PPARα), and sirtuin 1 (Sirt1). Yang and co-authors demonstrated that the miR-22-5p mimic could promote hepatic steatosis, while the miR-22-5p inhibitor effectively reduced triglyceride levels [49]. 

Resalis Therapeutics has developed an antago-miR-22-5p using locked nucleic acid (LNA) technology to design an antimiR named RES-010. Resalis Therapeutics reported that RES-010 restores the regulation of lipid biosynthesis, transforms white adipose tissue into brown adipose tissue, promotes metabolic rewiring towards higher energy expenditure, reduces and prevents hepatic steatosis, fibrosis, and inflammation, and induces weight loss. RES-010 is being developed for obesity and metabolic-associated fatty liver disease (MAFLD), as well as its progressive disease state, metabolic-associated steatohepatitis (MASH) [68]. The company plans to initiate a Phase 1 clinical study evaluating the safety and efficacy of RES-010 in 2024 [76]. Interestingly, a GaINAc conjugated version of RES-010, named RES-020, is in development for the treatment of obesity [77].

### 3.4. microRNA-103a-3p and microRNA-107

Other potential therapeutic targets identified for NAFLD/NASH include miR-103a-3p and miR-107 (Table 1). The sequences of these two microRNAs differ by only one nucleotide: the penultimate one is a guanosine (G) in miR-103a-3p, while it is a cytosine (C) in miR-107.

Initially, these two miRNAs were reported to be upregulated in the livers of obese mice [78]. Intriguingly, caveolin-1, a crucial insulin receptor regulator, appears to be specifically targeted by miR-103/107. It has been demonstrated that caveolin-1 is upregulated in adipocytes after miR-103/107 inactivation [60]. This is associated with the stabilization of the insulin receptor, improved insulin signaling, reduced adipocyte size, and enhanced glucose uptake as a result of insulin stimulation. In this way, miR-103/107 silencing leads to improvements in glucose homeostasis and insulin sensitivity [59].

In light of this, Regulus Therapeutics Inc. developed an anti-miR-103/107 oligonucleotide, AZD4076 (RG-125). This oligonucleotide is an RNA therapeutic that uses the conjugation of N-acetylgalactosamine (GalNAc) for the selective delivery to hepatocytes of anti-miR-103/107. GalNAc is a ligand with high affinity for the hepatocyte-specific asialoglycoprotein receptor (ASGR), involved in clathrin-mediated endocytosis [79].

In 2015, the first in-human phase 1 study of AZD4076 for NASH began. It is a randomized, single-blind, placebo-controlled study designed to assess the safety, tolerability, and pharmacokinetics of AZD4076 tetracosasodium after the administration of a single-ascending dose to healthy male subjects (Study identifier, D5590C00001; ClinicalTrials.gov identifier, NCT02612662). In 2016, another randomized, single-blind, placebo-controlled study was started, to assess the safety, tolerability, pharmacokinetics, and pharmacodynamics of AZD4076 after multiple-ascending dose administration in NAFLD patients with type 2 diabetes (Study identifier, D5590C00002; ClinicalTrials.gov identifier, NCT02826525). The original estimated enrollment was 51, but only 14 subjects were enrolled. RG-125 development was stopped in phase II in 2017 after serious adverse events were reported for one of the other miRNA-targeting agents under clinical development by the company alliance behind RG-125 [80].

## 4. Formulation Strategies to Improve miRNA-Based Therapies

As with other therapeutic modalities, the success of an oligonucleotide drug is dictated by the ability to specifically target the tissue and also by its pharmacokinetic behavior [81]. Oligonucleotide therapeutics comprise mainly drugs using antisense oligonucleotides (ASOs) [82,83] or using interference RNA (iRNA), such as small-interfering RNAs (siRNAs) [84] and miRNA [69], and also aptamers [85]. The advantage of this modality is that the pharmaceutical characteristics could be independently optimized to achieve improved pharmacokinetic (distribution or delivery) and pharmacodynamic (target regulation) properties in the treatment [81]. Oligonucleotides are chemically modified to optimize delivery into the target cell by adding sugars, bases, phosphate backbone, other nucleic acids, and other modifications on the ligand parts of 3′-End and 5′-End [67,81], predicting absorption, distribution, metabolism, and excretion [81].

An overreliance on a one-target therapy for multigenic complex diseases, such as HCC, would be challenging, although miRNA-based therapies could target multiple genes, increasing the effectiveness of the treatment [86]. However, RNA delivery has many issues, including avoiding the action of endo- and exonucleases, especially ribonucleases, which are abundant in vivo, increasing the half-life in the bloodstream and allowing the treatment to reach its specific tissue target [67], avoiding off-target effects. Therefore, the most concerning issue regarding the use of synthetic oligonucleotide therapy is overcoming its inherent characteristics. They are large, negatively charged molecules that do not easily penetrate the cell membrane, as lipophilic small drugs do; miRNAs could also activate Toll-like receptors and trigger inflammation. Therefore, improving their ability to reach, and effectively increase the cellular uptake of, the target host cell is essential in ensuring the success of gene therapy [87].

In addition to the chemical modifications that could be performed to improve stability, delivery, and cellular penetration, the modality of the delivery systems is also important. There are two basic delivery systems: viral and non-viral. The established vehicles for gene therapy are viral vectors, but they are prone to mutations and are more toxic and immunogenic in vivo [88]. Therefore, in this scenario, non-viral vehicles, such as liposomes and nanoparticle-based delivery, are attracting attention.

Currently, the available clinical trials for liver diseases use subcutaneous injection, delivering oligonucleotide drugs in a conjugation molecule with GalNAc, with chemical modifications, liposomes, or lipid-based nanoparticles (Table 1). Initially, the first way to solve the delivery problem was to exploit the human asialoglycoprotein receptor (ASGPR), which is expressed in hepatocytes. The addition of a trimer of N-acetylgalactosamine (GalNAc) to the oligonucleotide forms the biomolecule conjugation GalNAc, which binds to the ASGPR and is taken up to the cell by the endosome action [87]. Another chemical modification used is the locked nucleic acid (LNA), also known as the bridge nucleic acid, which is the connection (bridge) between the 2′-O,4′-C methylene linkage, conferring thermodynamic stability and enhanced nucleic acid recognition [89]. Other chemical modifications in miRNA include the addition of phosphorothioate bonds to the backbone of the oligonucleotide, conferring extra stabilization, protecting it from the action of the nucleases, and increasing its half-life in the bloodstream [88].

Another delivery strategy, and the most promising one, is the use of nanomolecules such as liposomes, lipid-based nanoparticles, and the multifunctional envelope nanodevice (MEND), all of which have been used to deliver miRNA into hepatocytes in vivo, thus limiting HCC development [90,91,92]. Daige and colleagues showed that the therapeutic miR-34a mimic, delivered by a liposome system in rodents, mainly reached the liver and the tumor as target tissue and was able to reduce the tumor weight and increase p53 expression levels without evidence of any immune response in mice nor in human whole-blood samples, as tested by the presence of cytokines [90]. A phase I clinical trial, conducted by an American group in 2016, tested the synthetic version of the miR-34a mimic (MRX34) in several tumor solid types, including HCC, encapsulated in a liposomal nanoparticle. The overall response was mainly stable disease, and one patient was in partial remission at the end of the study [91].

In addition, it has been noted that poly (ethyleneglycol) (PEG) is broadly used to stabilize lipid-based nanoparticles to solve the issue of the blood circulation time by preventing aggregation with other negatively charged serum compounds and the treatment clearance by the endosomal system. However, this PEGylation drastically decreases cellular uptake and impairs target tissue delivery [93]. To solve this problem, Sato et al. produced a synthetic multifunctional envelope nanodevice (MEND), which overcame this problem by the addition of a pH-sensitive cationic lipid, YSK05; this YSK05-MEND complex was able to escape from the endosomes and increase cellular uptake efficiently [92].

Other less exploited delivery strategies include the use of polymeric vectors, such as jetPEI (RosVivo Therapeutics Inc.), which is in development for use in liver disease, but its effectiveness and toxicity are not fully understood. In addition, some interesting in vitro studies have shown that the use of extracellular vesicles (EVs) is promising [94,95,96]. An Italian group developed an EV-encapsulated miR-125b produced in genetically modified mesenchymal stromal cells; in an in vitro environment, it was able to reduce HCC cell proliferation [94]. Pomatto et al., in another Italian study, tested the efficiency of EVs isolated from human plasma and engineered with miR-31 and miR-451a, transfecting them to the target cell in vitro by electroporation. They showed that this method of transfection did not alter the content of the EVs, which were effectively delivered to the host cells, and inhibited the anti-apoptosis activation pathway, increasing HCC cell death [96].

In summary, the best choice for improving miRNA-based therapies and ensuring a good treatment outcome is the combination of chemical modifications, to achieve miRNA stability, and the right drug carrier, to deliver it to the target inside the cell. The next results of the current available clinical trials will better guide researchers in paving the way for the treatment of HCC patients.

## 5. Challenges and Limitations of miRNA-Based Therapies

Many miRNAs can downregulate genes involved in HCC development, driving its progression from MASH and other chronic liver diseases. Thus, the transcriptional regulation of specific miRNAs could clinically impact multiple significant targets, representing promising therapeutic opportunities. This approach could be combined, for example, with transarterial chemoembolization (TACE) to improve treatment effectiveness. However, modifying miRNA expression can lead to unexpected effects due to its influence on multiple downstream targets [64]. Furthermore, the pharmacological application of mimics and antimiRs may have some significant drawbacks that require additional caution in their clinical application, e.g., low cellular uptake, instability of structure, and the degradation by nucleases [97]. Moreover, to exert a biological effect, they require supraphysiological concentrations that can saturate the RISC complex, thus also causing unwanted off-target effects [16,98]. Indeed, this problem has been resolved in recent years thanks to the new nanotechnology that allows precise delivery to a single tissue or cell type, as has been demonstrated using nanoparticles combined with engineered proteins to successfully deliver miRNAs to acute myeloid leukemia cells [99]. In the future, these new technologies could be effectively adapted for hepatic miRNA delivery, avoiding long-term safety concerns.

## 6. Conclusions

Since their discovery about thirty years ago, our understanding of miRNA functions in the regulation of physiological and pathological processes has significantly advanced, offering new possibilities for diagnostics and therapeutic interventions in multifactorial chronic liver diseases like MASH and HCC. The standardization of circulating miRNA expression profiles as non-invasive biomarkers for MASLD/MASH and HCC diagnostics is currently being evaluated and could soon become routine in clinical practice after validation in larger cohorts of patients.

miRNAs also show great promise as therapeutic agents for the treatment of MASLD/MASH and prevent tumor progression into HCC. The high perfusion rate and discontinuous sinusoidal endothelium of the liver may facilitate the hepatic uptake of miRNA mimics and inhibitors, as well as the abundance of receptors on the hepatocyte membrane. However, before miRNA-based therapies can be used in clinical practice, several challenges must be faced and resolved. For example, to avoid safety issues and side effects, the complete characterization of miRNAs of interest is necessary and can be achieved by understanding the interaction networks between miRNAs and their targets, e.g., mRNAs, protein factors, and other non-coding RNAs. Another concern is the efficient and specific delivery of miRNA mimics or inhibitors to hepatocytes. In conclusion, miRNA-based therapies are still in their early stages of development, and further research is warranted, to develop effective treatment options and to determine whether their use in clinical practice will be feasible in the future.

## Figures and Tables

**Table 1:** Clinical trials and major preclinical studies evaluating miRNA-based therapies for MASLD/MASH.

Target miRNA	Strategy	Accession miRbase Number	Sequence	Target Genes	Commercial Name and Company	Delivery System	References
miR-103a-3p	Inhibition	MIMAT0000101	AGCAGCAUGUACAGGGCUAUGA	Caveolin-1	AZD4076 (RG-125) (Regulus Therapeutics Inc.)	Biomolecule conjugation (GalNAc)	NCT02612662 NCT02826525
miR-107	Inhibition	MIMAT0000104	AGCAGCAUUGUCAGGGCUAUCA				
miR-22-5p	Inhibition	MIMAT0004495	AGUUCUUCAGUGGCAAGCUUUA	Peroxisome proliferative-activated receptor (Pgc-1α) Peroxisome proliferator-activated receptor α (PPARα) Sirtuin 1 (Sirt1)	RES-010 (Resalis Therapeutics)	Lipid-based nanoparticles	[68]
miR-132-3p	Inhibition	MIMAT0000426	UAACAGUCUACAGCCAUGGUCG	Phosphatase and tensin homolog (Pten) Forkhead box O3 (FOXO3) Sirtuin 1 (Sirt1)	Anti-miR-132-3p (Regulus Therapeutics Inc.)	Biomolecule conjugation (GalNAc)	[69]
miR-10b-5p	Mimic	MIMAT0000254	UACCCUGUAGAACCGAAUUUGUG	Transcription factor Krüppel-like factor 11 (KLF11)	RSVI-301 (RosVivo Therapeutics Inc.; Eli Lilly)	Subcutaneous injection with *jetPEI* agent transfection	[56]
